# Red Blood Cell Exchange in a Patient With Extramedullary Hematopoiesis and Cor Pulmonale Secondary to Beta Thalassemia

**DOI:** 10.7759/cureus.13638

**Published:** 2021-03-01

**Authors:** Pallavi Kopparthy, Amar H Kelkar, Kunal Aggarwal, Samantha De Filippis, Brad Fletcher

**Affiliations:** 1 Division of Hematology & Oncology, Department of Medicine, University of Florida College of Medicine, Gainesville, USA; 2 Medical Education, Saint George's University School of Medicine, True Blue, GRD; 3 Medical Education, University of Medicine and Health Sciences, Camps, KNA

**Keywords:** beta thalassemia, red blood cell exchange, extramedullary hematopoiesis, hbf, cor pulmonale, hemoglobinopathy, packed red blood cell transfusion

## Abstract

Rationale: Beta thalassemia is a congenital defect in the production of the beta globin chain. Patients with beta thalassemia major will have higher levels of hemoglobin F (HbF), which is suboptimal in releasing oxygen to tissue. Herein, we describe the use of red blood cell (RBC) exchange transfusion, a therapy typically used in sickle cell patients, in the management of a patient with beta thalassemia with extensive extramedullary hematopoiesis and elevated levels of HbF.

Patient concerns: A 34-year-old male of mixed African American and Southeast Asian descent with a known history of beta thalassemia major presented with progressive dyspnea on exertion with marked fatigue.

Diagnoses: The patient was transferred to our facility for management of acute hypoxemic, hypercapnic respiratory failure associated with cor pulmonale.

Interventions: The patient was initially managed with non-invasive positive pressure support ventilation (NIPPV) and intravenous diuresis. Hydroxyurea and epoprostenol nebulization were added to his treatment regimen; however, he progressively became more unstable, necessitating inotropic support. With extramedullary hematopoiesis leading to mass-like effect on critical organs and very high HbF (96%) thought to contribute to his presentation, red blood cell exchange transfusion was initiated once the blood pressure stabilized.

Outcomes: The patient clinically improved, and was discharged home within a week on supplemental oxygen by nasal cannula and long-term red blood cell exchange.

Lessons: We postulated that significantly elevated HbF contributed to the patient’s chronic hypoxia and subsequent respiratory complications. Based on the patient's clinical improvement following the intervention, we believe that RBC exchange transfusion could be considered in the management of beta thalassemia patients with significantly elevated levels of HbF.

## Introduction

Thalassemia is a form of microcytic anemia where there is quantitatively decreased synthesis of structurally normal alpha or beta globin proteins. Beta thalassemia refers to diminished beta globin production caused by mutations affecting one or both of the beta globin genes. It is prevalent among various populations around the world, including people of African American, Asian Indian, and Southeast Asian descent, each with their own unique mutations [1]. Beta thalassemia major, the most severe form of beta thalassemia, also known as Cooley's anemia, Mediterranean anemia, and transfusion-dependent thalassemia, occurs when there are multiple mutations or deletions of beta globin alleles, disrupting beta globin chain production and causing hemoglobin A (HbA; ɑ2β2) to be significantly diminished or entirely absent [2].

Symptoms of beta thalassemia major are usually evident within the first six to 12 months of life. Fetal hemoglobin (HbF; α2γ2) levels are often upregulated as a compensatory response and remain high throughout the lifespan of these patients. Patients are also, often, transfusion-dependent. In the absence of adequate red blood cell (RBC) transfusions, the infant may experience failure to thrive or a variety of other clinical findings, such as a predisposition to fractures due to erythroid expansion causing cortical bone thinning and osteopenia, growth retardation, progressive hepatosplenomegaly, gallstone formation, and cardiac disease [3-4]. An individual diagnosed early in life who has access to aggressive therapy, including hypertransfusion, iron chelation, and, in some cases, hematopoietic cell transplantation, may have substantially fewer sequelae and improved survival into the fourth, fifth, and sixth decades. Herein, we present the case of a 34-year-old male with complications from beta thalassemia major with extensive extramedullary hematopoiesis and 96% HbF levels, treated with RBC exchange transfusion as a means to improve oxygen delivery and minimize iron overload.

## Case presentation

A 34-year-old male of mixed African American and Southeast Asian (Cambodian) descent presented to a regional hospital with progressive dyspnea on exertion. He had a known history of beta thalassemia major (β0/β0) previously managed with splenectomy and jaw surgery due to extramedullary hematopoiesis. The patient had been lost to follow-up after the patient moved away from the immediate area and the patient reported limited contact with medical care for approximately 15 years. His last RBC transfusion was approximately one year prior to this hospitalization, but it was noted that he had very few blood transfusions during his adult life and was never placed on an exchange transfusion protocol.

Physical examination on presentation to our facility was significant for labored breathing, slight stature, proximal muscle wasting, frontal bossing, fine crackles and wheezing at the lung bases, and sinus tachycardia. Sensorium, including orientation, was fully intact. The patient reported marked fatigue with generalized weakness, but no focal deficits or cranial nerve abnormalities were observed.

**Figure 1:**
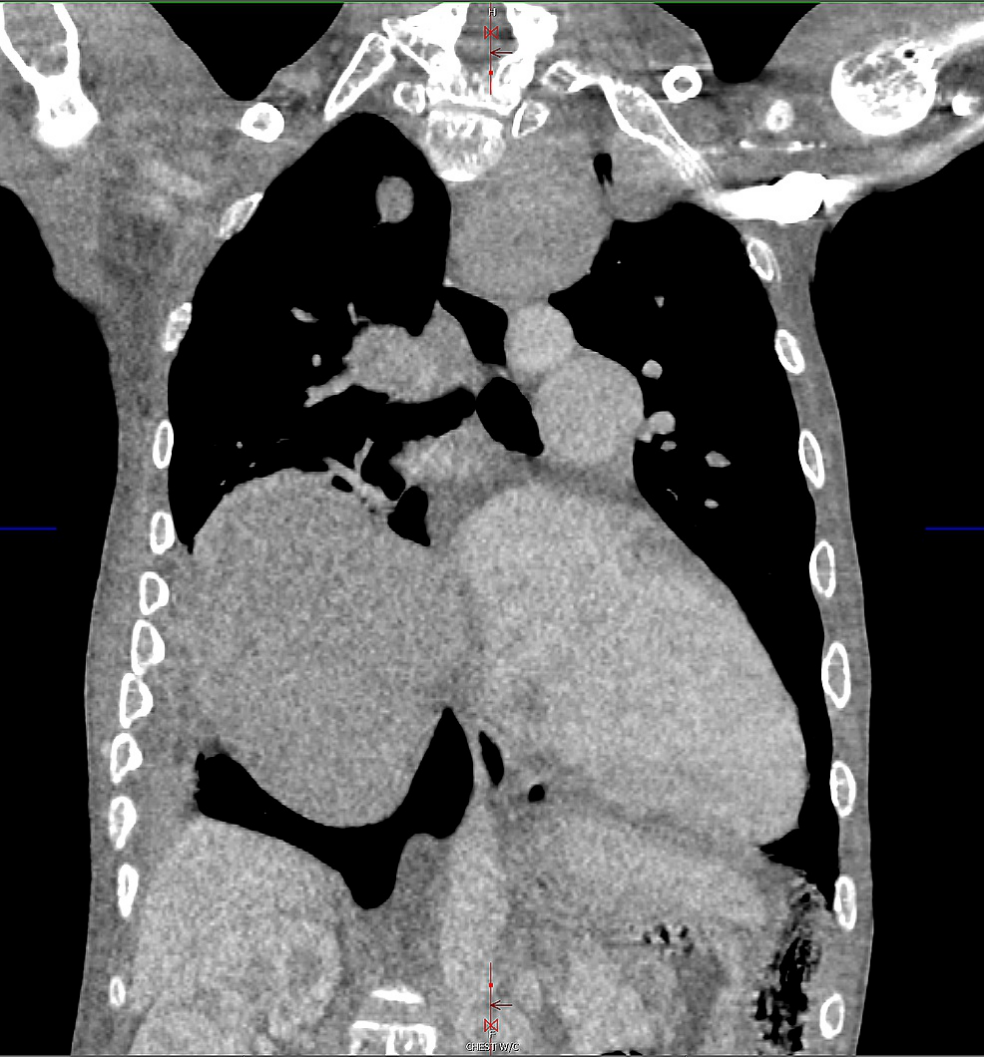
Computed Tomography of the Thorax: Coronal plane view demonstrates multiple soft tissue masses in the left hemithorax, as well as bilateral in the posterior paraspinal region, with the largest lesion on the right measuring over 9 cm in size.

**Figure 2:**
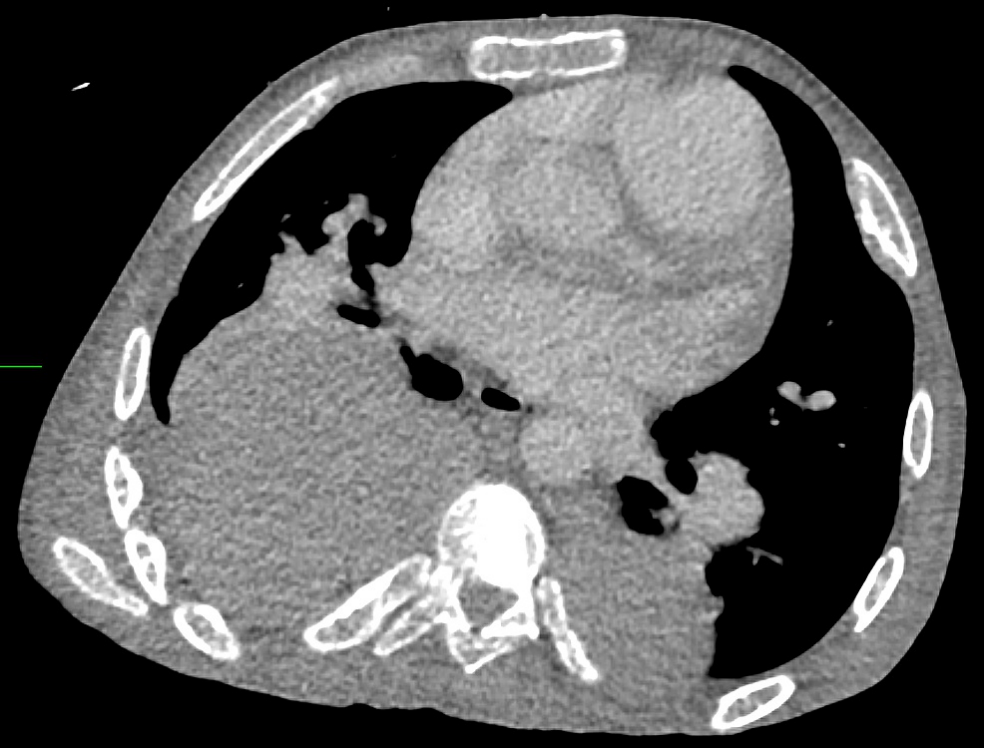
Computed Tomography of the Thorax: Axial plane view demonstrating posterior paraspinal masses, particularly a large right posterior paraspinal mass.

**Figure 3:**
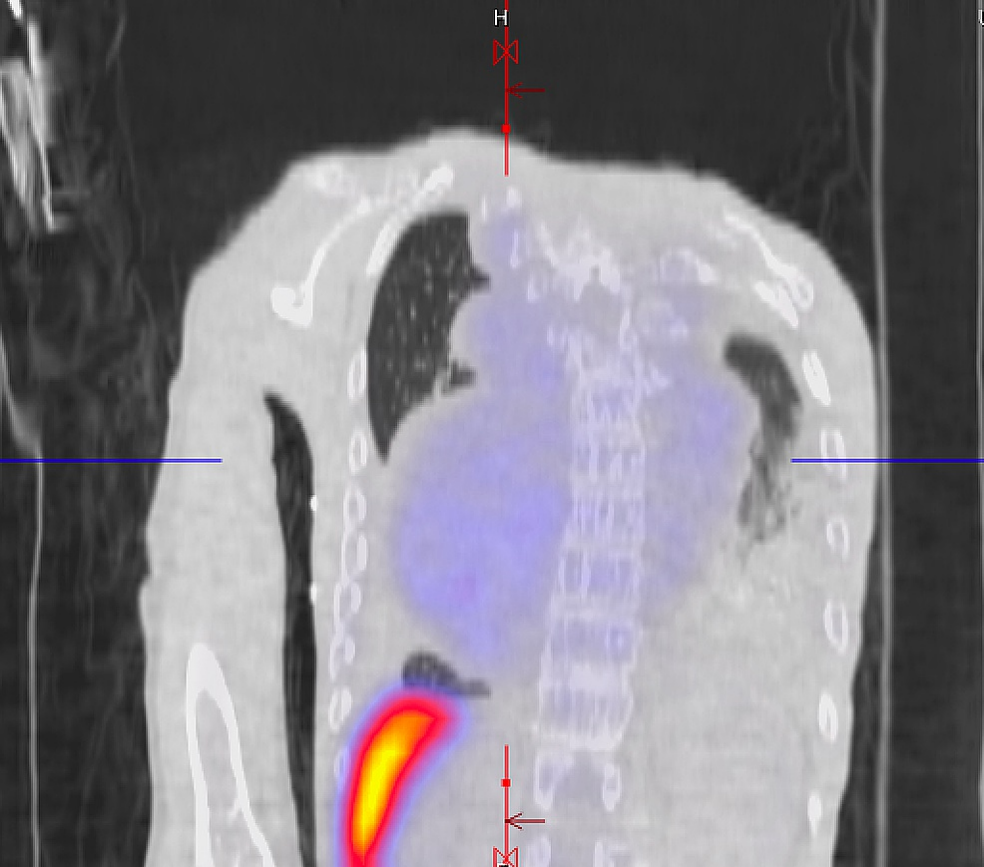
Nuclear Medicine Bone Marrow Scan following the intravenous administration of 15 mCi of 99m technetium-filtered sulfur colloid, and a 20 minute delay, whole body computed tomography images as well as single-photon-emission computed tomography (SPECT/CT) of the chest were performed, where colored areas correspond to uptake of the radiotracer. SPECT/CT images in the coronal plane view depict paraspinal conglomerates at the level of the thoracic spine with associated increased uptake (light blue), suggestive of extramedullary hematopoiesis, compressing the thoracic cavity without involvement of the lung parenchyma, and widespread marrow expansion, with a markedly enhanced liver capsule (red-yellow).

**Figure 4:**
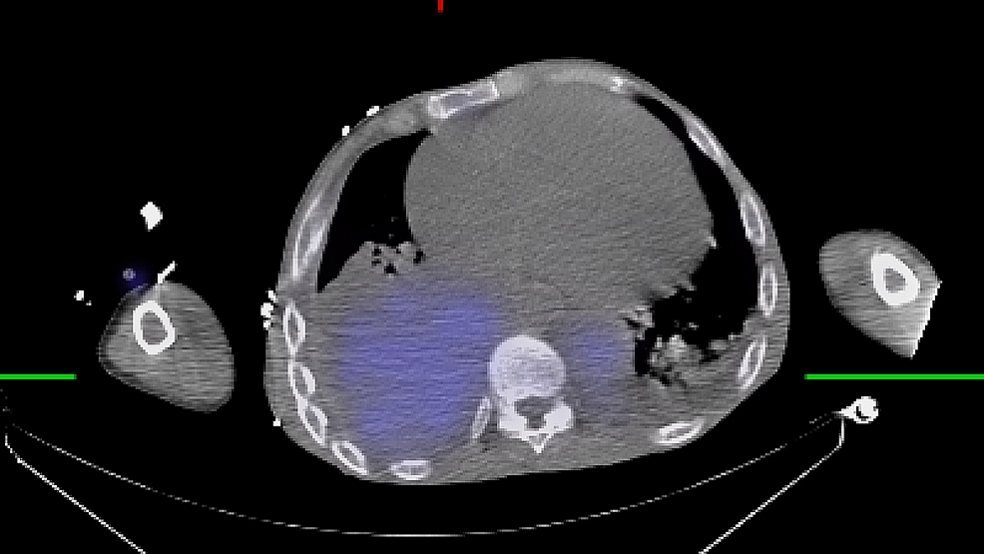
Nuclear Medicine Bone Marrow Scan following the intravenous administration of 15 mCi of 99m technetium-filtered sulfur colloid, and a 20 minute delay, whole body computed tomography images as well as single-photon-emission computed tomography (SPECT/CT) of the chest were performed, where colored areas correspond to uptake of the radiotracer. SPECT/CT images in the axial plane view of the thorax highlighting posterior paraspinal extramedullary hematopoiesis in the thoracic cavity (light blue).

**Figure 5:**
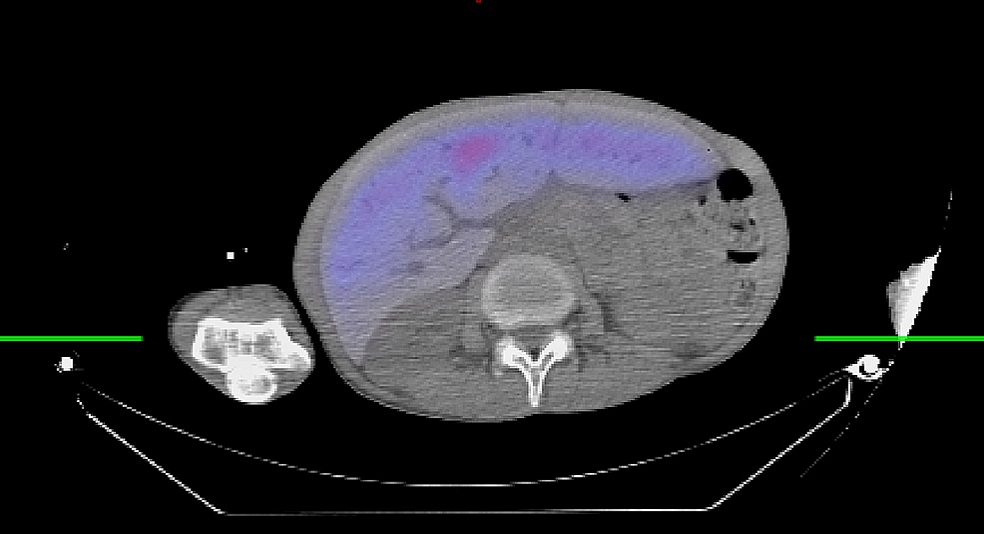
Nuclear Medicine Bone Marrow Scan following the intravenous administration of 15 mCi of 99m technetium-filtered sulfur colloid, and a 20 minute delay, whole body computed tomography images as well as single-photon-emission computed tomography (SPECT/CT) of the chest were performed, where colored areas correspond to uptake of the radiotracer. SPECT/CT images in the axial plane view of the abdomen showing extramedullary hematopoietic activity (light blue) likely overlying background physiologic uptake in the liver capsule.

Early in the hospital course, the patient was transferred to our tertiary facility for management of acute hypoxemic, hypercapnic respiratory failure. This presentation was complicated by cor pulmonale secondary, presumed to be secondary to beta thalassemia, and required prolonged high-pressure face mask ventilation. The initial laboratory values are presented in Table 1. Initial hemoglobin electrophoresis revealed HbF 96% and HbA2 4%, HbA or hemoglobin S (HbS) were unmeasurable. Transthoracic echocardiography at the outside facility revealed a preserved ejection fraction of 55%, severe tricuspid regurgitation, severe dilatation of the right atrium and right ventricle, patent foramen ovale, and right ventricular systolic pressure of 76 mmHg. Iodine contrast-enhanced computed tomography imaging of the thorax was negative for pulmonary emboli and showed “multiple soft tissue masses in the anterior left hemithorax, as well as bilateral in the posterior paraspinal region, largest lesion on the right measuring over 9cm in size” (Figure 1, 2). During the course of the evaluation, a biopsy was planned to confirm the diagnosis of extramedullary hematopoiesis but was changed to Nuclear Medicine Bone Marrow Scan due to technical difficulty and concern for unnecessary risk (Figure 3-5).

**Table 1:** Laboratory Values: (A) Hematology; (B) Arterial Blood Gas; (C) Blood Chemistry. g, grams; dL, deciliters; WBC, white blood cells; mm^3^, cubic millimeters; HbF, hemoglobin F; HbA_2_, hemoglobin A_2_; HbA, hemoglobin A; HbS, hemoglobin S; pCO2, partial pressure of carbon dioxide; mmHg, millimeters of mercury; mmol, millimoles; L, liters; mg, milligrams; pg, picograms; IU, international units; ng, nanograms; mL, milliliters

	Measured Value	Reference Value
Hemoglobin	10.3 g/dL	13.0-16.5 g/dL (male)
White Blood Cell (WBC)	5150/mm^3^	4000-10000/mm^3^
HbF	96%	0.8-2.0%
HbA_2_	4%	2.0-3.0%
International normalized ratio	1.7	0.8-1.1
Platelet count	309,000/mm^3^	150,000-450,000/mm^3^
*WBC count manually re-evaluated due to high numbers of nucleated red blood cells *HbA or HbS were not measured		

	Measured Value	Reference Value
pH	7.22	7.35-7.45
pCO2	80.9 mmHg	35.0-45.0 mmHg
Carboxyhemoglobin	3.4%	0.0-2.0%
Methemoglobin	2.3%	0.0-0.9%

	Measured Value	Reference Value
Sodium	139 mmol/L	136-145 mmol/L
Potassium	6.1 mmol/L	3.3-5.1 mmol/L
Chloride	102 mmol/L	98-107 mmol/L
Bicarbonate	28 mmol/L	22-30 mmol/L
Blood urea nitrogen	24 mg/dL	6-21 mg/dL
Serum creatinine	0.44 mg/dL	0.51-1.18 mg/dL
B-natriuretic peptide level	742 pg/mL	< 100 pg/mL
Aspartate aminotransferase	45 IU/L	0-37 IU/L
Alanine aminotransferase	23 IU/L	0-50 IU/L
Total bilirubin	4.7 mg/dL	0.0-1.0 mg/dL
Direct bilirubin	1.4 mg/dL	0.0-0.2 mg/dL
Alkaline phosphatase	78 IU/L	40-150 IU/L
Ferritin	1448.0 ng/mL	24.0-336.0 ng/ml

The patient was initially managed with pressure support ventilation and diuresis with intravenous furosemide, which were tolerated with good urine output. Hydroxyurea was added to try to reduce extramedullary hematopoiesis and the associated masses, initially with 1 gram daily, which was then increased to 1.5 grams daily. Though hydroxyurea is known to increase HbF, it was added to the regimen in an effort to reduce extramedullary hematopoiesis and the associated masses. Epoprostenol nebulization was used to manage pulmonary hypertension with plans for right heart catheterization once the patient was more stable. However, the patient evolved poorly, with the need for inotropic support with infusional milrinone.

Once the patient’s blood pressure stabilized, automated RBC exchange transfusion was planned due to the high baseline hemoglobin level. A separate vascular catheter was placed, for the purposes of exchange transfusion, and four units of donor-matched RBCs were transfused via automated exchange transfusion with subsequent hemoglobin electrophoresis showing HbF 55%, HbA 42%, and HbA2 3%. Following this procedure, the patient clinically improved, coming off pressure-supported ventilation to high-flow nasal oxygen as well as milrinone, and was weaned to nasal cannula oxygen within one week. He was discharged with plans for monthly outpatient RBC exchange transfusions.

## Discussion

Untreated, beta thalassemia major has high mortality with as many as 85% of untreated individuals dying by the age of five years, mostly from cardiovascular complications [5]. Patients can have symptoms related to severe anemia, including high-output heart failure, failure to thrive, infection, and expanding sites of extramedullary hematopoiesis, including skeletal abnormalities of the face and long bones [3-4]. An increased incidence of cerebral thrombosis, venous thromboembolism, and pulmonary hypertension has been reported in beta thalassemia major and beta thalassemia intermedia following splenectomy, and these risks should be considered before splenectomy [6-7]. Our patient had a history of splenectomy but was lost to follow-up for more than 15 years and presented with extensive intrathoracic extramedullary hematopoiesis and cor pulmonale.

Management of patients with beta thalassemia major involves a multidisciplinary care approach with close monitoring of iron stores and disease complications. RBC transfusion has been the mainstay in the management of beta thalassemia major to suppress ineffective erythropoiesis. A lifelong blood transfusion program to maintain a pretransfusion Hb level between 9-10 g/dL has been demonstrated to suppress bone marrow expansion while minimizing transfusional iron loading [8]. Red blood cell exchange is performed to reduce the risk of iron overload in chronically transfused patients, most often in sickle cell disease [9]. Hypocalcemia, typical blood transfusion risks, and coagulopathy with bleeding are the most notable adverse effects associated with this therapy. In cases of transfusion-dependent beta thalassemia, such as in this case where it was leading to severe extramedullary hematopoiesis, the exchange transfusion protocol was used to increase oxygenation and suppress extramedullary hematopoiesis.

Apart from transfusions, iron chelation to prevent and treat iron overload is another mainstay of beta thalassemia treatment [10]. A complete iron load evaluation includes at least serum ferritin every three months, annual liver iron concentration (LIC) by magnetic resonance imaging R2 starting at age five, and annual cardiac iron T2* starting between eight to 10 years of age. Iron chelation therapy with subcutaneous deferoxamine, oral deferasirox, or deferiprone is initiated when serum ferritin levels reach around 1,000-1,500 ng/mL following approximately 12 months of scheduled transfusions or approximately 20 units of blood. Chelation is adjusted to maintain a ferritin <1,000-1,500 ng/mL, a LIC of 2-7 mg of iron/g dry weight, and a cardiac T2* >20 ms [11]. However, these treatment strategies do not address the cardiopulmonary complications that were evident in our patient. Phosphodiesterase type 5 inhibitors (e.g., sildenafil) and the endothelin receptor antagonist (e.g., bosentan) have been reported to be used to treat pulmonary hypertension in patients with beta thalassemia [12]. Additionally, allogeneic hematopoietic stem cell transplantation (HSCT) has shown promise with regards to being a curative therapy for beta thalassemia, but has been limited by donor availability [13]. Several novel targeted therapies such as luspatercept, gene therapies, and gene editing techniques using the CRISPR/cas9 system were in development at the time of managing this patient. Given the age, disease severity, and transfusion-dependence, our management likely would have changed to include luspatercept and potentially one of these gene therapies [13-14].

Etiologies of cardiopulmonary complications such as group 5 pulmonary hypertension and subsequently cor pulmonale in beta thalassemia are believed to be multifactorial [15]. These issues could be due to hemolysis-induced low arginine and nitric oxide bioavailability, hypercoagulability secondary to splenectomy, and iron deposition in the lung, liver, or heart from blood transfusions [16]. Another possible etiology for such complications, especially in untreated beta thalassemia major, could be due to persistently elevated HbF, sometimes up to 100%. Hemoglobin F shifts the hemoglobin-oxygen dissociation curve towards higher oxygen affinity, thus inducing functional anemia resulting in persistent tissue hypoxia [17]. It is known that chronic hypoxia can lead to pulmonary hypertension and pulmonary vascular remodeling [16]. Our patient had HbF 96% on presentation and had a hemoglobin level greater than 9 g/dL. We, therefore, proceeded with RBC exchange transfusion to increase available HbA and reduce levels of HbF with the goal of improving tissue perfusion to accelerate recovery and improve respiratory status, while reducing the risk of iron overload. Red blood cell exchange has been commonly used in sickle cell disease to replace HbS with HbA and improve tissue oxygenation [9]. To our knowledge, this is the first published case where RBC exchange transfusion has been used to improve oxygenation in patients with beta thalassemia major. A thorough review of the literature revealed two other cases where RBC exchange was used to treat leg ulcerations in beta thalassemia intermedia [18-19]. The impetus for red blood cell exchange transfusion was to add healthy RBCs, and to a lesser extent reduce the high levels of circulating HbF, thus improving tissue oxygenation and reducing the oxygen demand contributing to extramedullary hematopoiesis. Long-term, this technique also reduces the incidence of iron overload and subsequent chelation therapy, which is why red blood cell exchange has been shown to be cost equivalent to simple transfusion [20]. Our patient was concomitantly treated with epoprostenol for pulmonary hypertension and was discharged on tadalafil and a close follow-up with hematology who eventually put him on a chronic RBC exchange program.

## Conclusions

We believe that RBC exchange transfusion should be considered in patients with beta thalassemia major, extramedullary hematopoiesis, and high levels of HbF, particularly in cases with higher baseline Hb between 9-10. Apart from improving oxygenation, preventing the development of medical complications driven by chronic hypoxia in patients with thalassemia, RBC exchange transfusion also protects against iron overload and is more efficient relative to transfusions. In addition, HSCT and luspatercept could be considered, but due to limited donor availability and financial and therapy-related toxicity, not everyone is a potential candidate. However, RBC exchange transfusion could still be considered in such patients. We hope that our work will encourage clinicians to continue to re-evaluate current standards of care for patients with beta thalassemia and spur additional research in this area.
